# Malaria and dyserythropoiesis: a mini review

**DOI:** 10.3389/fcimb.2025.1679337

**Published:** 2025-09-05

**Authors:** Fang-Fang Liu, Ke Li

**Affiliations:** ^1^ Department of Pathology, The Central Hospital of Wuhan, Tongji Medical College, Huazhong University of Science and Technology, Wuhan, China; ^2^ School of Life and Health Sciences, Hainan Province Key Laboratory of One Health, Collaborative Innovation Center of One Health, Hainan University, Haikou, Hainan, China

**Keywords:** malaria, anemia, dyserythropoiesis, ineffective erythropoiesis, bone marrow niche, hemozoin, inflammatory cytokines

## Abstract

Malaria associated anemia is increasingly recognized as a consequence not only of red cell destruction but of profound, parasite driven disruption of erythropoiesis within the bone marrow niche. Here, we synthesize recent *in vitro*, ex vivo, clinical and postmortem studies to construct a unified mechanistic framework in which four interlocking pathways converge to produce dyserythropoiesis. First, a cytokine storm dominated by IL-6, TNF-α, IFN-γ and MIF suppresses erythropoietin synthesis, upregulates hepcidin and diverts erythroid progenitors toward myeloid fate via destabilization of GATA-1. Second, hemozoin crystals catalyze Fenton chemistry and lipid peroxidation, generating 4-hydroxynonenal adducts that cripple GATA-1 and trigger mitochondrial apoptosis of erythroblasts. Third, *Plasmodium* parasites preferentially infect orthochromatic erythroblasts, prolonging a 10-day gametocyte maturation cycle beyond the host’s 3–4-day enucleation window and releasing extracellular vesicles that arrest terminal differentiation. Fourth, hemozoin-laden macrophages remodel erythroblastic islands, precipitating local iron restriction and sustained oxidative stress. Together these processes create a “developmental sanctuary” that favors parasite persistence while crippling host erythropoiesis. We also highlight emerging single-cell and spatial-omics technologies, together with 3-D bone-marrow organoids, as platforms for dissecting spatiotemporal parasite–host interactions and for testing niche-targeted therapies aimed at reversing ineffective erythropoiesis.

## 1 Introduction

Malaria, a major parasitic disease threatening global public health security, exhibits pathological complexity not only in the systemic inflammatory response during acute infection but also in the long-term impact of chronic anemia on patient quality of life. According to the World Health Organization’s 2024 World Malaria Report, there were an estimated 263 million malaria cases globally in 2023, with approximately 597,000 malaria-related deaths (Venkatesan, 2025). Severe anemia, a critical complication resulting from red blood cell destruction, significantly contributes to malaria’s severity and mortality, particularly in high-transmission regions. Children under five years of age and pregnant women represent particularly vulnerable groups. In high-transmission areas, malaria is one of the most common indications for blood transfusion, yet transfusion thresholds in severe malaria management remain uncertain (Ackerman et al., 2020). Although artemisinin-based combination therapies (ACTs) have significantly reduced acute-phase mortality, clinical observations reveal that a subset of patients experience persistent anemia even after parasite clearance (Eastman and Fidock, 2009; Fernandez-Arias et al., 2016; Ndour et al., 2017).

Classical theories attribute malarial anemia to three primary mechanisms: direct parasite lysis of infected red blood cells (RBCs); splenic hyper-removal of uninfected RBCs with impaired deformability and disturbances in iron metabolism coupled with insufficient erythropoietin (EPO) synthesis due to chronic inflammation (Looareesuwan et al., 1991; Dondorp et al., 1999). However, this framework is increasingly challenged by new evidence. For instance, The deposition of malaria pigments (hemozoin) in the bone marrow directly induces apoptosis of erythroid precursors and is significantly associated with ineffective erythropoiesis and dyserythropoiesis in bone marrow (Casals-Pascual et al., 2006; Lamikanra et al., 2009; Aguilar et al., 2014), while iron kinetic studies demonstrate that the decline in hemoglobin synthesis rate precedes RBC destruction (Pathak and Ghosh, 2016). Thus, ineffective erythropoiesis and dyserythropoiesis may be potential core drivers of malaria anemia, while the bone marrow is a “hidden battlefield”. Substantial progress in understanding malaria pathogenesis has stemmed from meticulous characterization of the bone marrow microenvironment. Intravital imaging confirms that immature *P. falciparum* gametocytes and *P. vivax* asexual/sexual stages colonize the bone marrow, forming a significant reservoir (Joice et al., 2014; Baro et al., 2017; De Niz et al., 2018). Single-cell transcriptomics in murine models reveal dysregulation of erythroid transcriptional networks (e.g., GATA1) in infected progenitors, contributing to impaired erythropoiesis (Silva-Filho et al., 2020). Additionally, parasite metabolites (e.g., hemozoin) and inflammatory cytokines (TNF-α, IFN-γ) disrupt hematopoietic niche function, triggering microenvironmental remodeling (Silva-Filho et al., 2020).

The regulatory role of host genetic background in anemia susceptibility cannot be overlooked. Polymorphisms in the ABO and Duffy blood group systems significantly influence disease progression: individuals with blood group O exhibit reduced *P. falciparum* PfEMP1-mediated cytoadhesion due to the absence of A/B antigens (Degarege et al., 2019), while Duffy antigen-negative populations (common in individuals of African descent) display innate resistance to *P. vivax* infection (Baird, 2022). Since blood group influences malaria susceptibility, the anemia status of individuals with different blood groups after contracting malaria may vary. This may also suggest that blood group molecules could potentially impact erythropoiesis. However, changes in the expression of blood group molecules might merely be a downstream manifestation of altered erythropoiesis. Overall, the pathology of malaria anemia may result from the interplay of host genetics, parasite virulence, and microenvironmental remodeling.

This mini-review aims to elucidate the bone marrow pathogenesis of malarial anemia, focusing on three core questions: 1) How parasites disrupt erythropoiesis through direct infection and paracrine effects; 2) How the inflammatory microenvironment and oxidative stress synergistically disrupt hematopoietic homeostasis; and 3) Prospects for developing novel therapeutic strategies targeting bone marrow pathways. By integrating clinical data, molecular biology evidence, and innovative model systems (e.g., bone marrow organoids), this study will provide a theoretical foundation for reversing ineffective erythropoiesis.

## 2 Cytokine-mediated suppression of erythropoiesis

To control parasitemia, host immune cells release a cascade of pro-inflammatory and anti-inflammatory cytokines, chemokines, growth factors, and other mediators. While these immune responses confer host protection, in this inflammatory state, multiple factors simultaneously suppress erythropoiesis by interfering with erythroid progenitor differentiation, disrupting iron metabolism homeostasis, and altering the hematopoietic microenvironment, thereby exacerbating anemia.

During the early stages of malaria, the cytokine IL-6 plays a protective role, stimulating the acute phase response and supporting immune cell differentiation (Gowda and Wu, 2018). Peripheral blood mononuclear cells (PBMCs) are likely the primary source of IL-6 during acute malaria (Aubouy et al., 2002). Studies in murine models indicate that interleukin-6 (IL-6) mediates protective immunity against the pre-erythrocytic stage of malaria by inducing interleukin-1β (IL-1β) and tumor necrosis factor-α (TNF-α), and enhances specific immunoglobulin G (IgG) antibodies to control blood-stage parasitemia during the erythrocytic phase (Pied et al., 1992). However, as the disease progresses, persistently elevated levels of IL-6, IL-1β, and others lead to systemic inflammation (Obeagu, 2024). Research in malaria infection models reveals that IL-6 regulates iron metabolism homeostasis through a dual mechanism: on one hand, it induces hepatic hepcidin synthesis via the JAK2-STAT3 pathway, resulting in decreased serum iron concentration (Wrighting and Andrews, 2006); on the other hand, it directly suppresses renal erythropoietin (EPO) expression (Jelkmann, 1998). Furthermore, elevated serum IL-6 levels in malaria patients positively correlate with anemia severity (Lyke et al., 2004).

Beyond IL-6, IL-1β also plays a crucial role in suppressing erythropoietic differentiation. This cytokine promotes PU.1 expression by activating the NLRP3 inflammasome (Pietras et al., 2016), while simultaneously disrupting the erythroid-myeloid differentiation balance through caspase-1-mediated proteolysis of GATA-1 (Tyrkalska et al., 2019). Indeed, similar to TNF-α, IL-1β promotes the acute inflammatory response at the onset of malaria infection, providing a first line of defense against invading pathogens (Penha-Goncalves, 2019). For example, IL-1β can synergize with IL-1α and TNF-α to enhance nitric oxide (NO) and IFN-γ production in murine malaria models (Rockett et al., 1994). However, sustained high-level production of IL-1β can induce anemia, a phenomenon also documented in numerous other disease models beyond malaria (Pascual et al., 2005).

Members of the interferon family, particularly IFN-γ, play a prominent role in inflammation-associated anemia. *Plasmodium* parasites and their byproducts, such as hemozoin, can amplify a robust inflammatory response by increasing TNF-α and IFN-γ (Giribaldi et al., 2004; Jaramillo et al., 2004). While these inflammatory mediators stimulate monocyte/macrophage activation and help control parasitemia during the early infection phase (Kremsner et al., 1995), substantial evidence confirms that their persistent overproduction can also significantly suppress erythropoiesis (Miller et al., 1989; Felli et al., 2005). IFN-γ promotes myeloid differentiation via the IRF1/PU.1 axis (Libregts et al., 2011). In TLR9-driven models of hemophagocytic lymphohistiocytosis (HLH), this cytokine not only suppresses bone marrow erythropoiesis but also limits compensatory splenic erythropoiesis (Canna et al., 2013).

Macrophage migration inhibitory factor (MIF) plays a distinct role in parasite infection-associated anemia. The malarial metabolite hemozoin induces monocytes to release MIF, which exerts synergistic inhibitory effects with TNF and IFN-γ (Martiney et al., 2000; McDevitt et al., 2015). Furthermore, parasite-secreted MIF homologs may amplify the suppression of uninfected erythroid progenitors (Ghosh et al., 2019). Additionally, studies show that MIF inhibits erythropoietin-dependent erythroid colony formation, as well as colony formation derived from multipotential (CFU-GEMM) and granulocyte-macrophage (CFU-GM) progenitor cells (Martiney et al., 2000; Chaiyaroj et al., 2004). It has been also demonstrated in murine studies that compared to wild-type controls, MIF-knockout mice infected with malaria develop less severe anemia, exhibit improved erythroid progenitor development, and display higher survival rates (McDevitt et al., 2006).

## 3 Oxidative damage effects of hemozoin

Hemozoin (Hz), a dark brown crystalline substance, is formed when malaria parasites detoxify toxic heme into insoluble β-hematin crystals during hemoglobin digestion within infected erythrocytes. Following erythrocyte invasion, *Plasmodium* parasites extensively ingest host hemoglobin, transport it to acidic digestive vacuoles for proteolytic degradation, and polymerize liberated heme into Hz via biomineralization. Clinical studies demonstrate that plasma Hz levels correlate significantly with anemia severity and reticulocyte suppression in malaria patients (Aguilar et al., 2014), while *in vitro* exposure to Hz directly inhibits erythropoiesis (Giribaldi et al., 2004; Skorokhod et al., 2010). Crucially, Hz accumulates in the bone marrow niche where it inflicts direct damage on erythroid hematopoiesis primarily through induction of oxidative stress and lipid peroxidation cascades.

### 3.1 Iron-mediated ROS generation and cellular consequences

The iron-rich crystalline lattice of Hz catalyzes robust Reactive Oxygen Species (ROS) production via Fenton chemistry (Schwarzer et al., 2003; Dumarchey et al., 2022). Studies have shown bone marrow Hz burden correlates positively with anemia severity in patients (Casals-Pascual et al., 2006; Aguilar et al., 2014). Experimental models confirm that Hz-exposed macrophages and erythroid precursors exhibit markedly elevated ROS levels, resulting in membrane lipid peroxidation, mitochondrial dysfunction, and direct suppression of erythroid progenitor proliferation and differentiation (Jaramillo et al., 2003; Shio et al., 2009; Cambos et al., 2010; Barrera et al., 2011). This oxidative milieu further activates pro-apoptotic pathways (notably caspase-3) to induce erythroblast apoptosis (Lamikanra et al., 2009; Skorokhod et al., 2010). Importantly, Hz-induced oxidative stress may trigger mitochondrial dysfunction, as elevated ROS levels are known to disrupt mitochondrial integrity in other pathological contexts (Zorov et al., 2014). This could potentially initiate a cycle of amplified oxidative damage and depletion of antioxidants such as glutathione (GSH) (Kotepui et al., 2023), though direct evidence linking Hz to mitochondrial electron transport chain disruption remains limited.

### 3.2 4-Hydroxynonenal as a cytotoxic effector

Hz-triggered peroxidation of polyunsaturated fatty acids (PUFAs) non-enzymatically generates 4-hydroxynonenal (4-HNE), a highly reactive lipid peroxidation end-product (Miller et al., 2005; Skorokhod et al., 2005). *In vitro* studies reveal that even low concentrations of 4-HNE potently inhibit BFU-E and CFU-E colony formation independently of apoptosis induction (Giribaldi et al., 2004; Skorokhod et al., 2010). Instead, 4-HNE imposes G0/G1 cell cycle arrest in erythroid progenitors and downregulates expression of GATA-1—a master transcriptional regulator of erythropoiesis—through covalent modification of its DNA-binding domain (Skorokhod et al., 2010). This dual action disrupts hemoglobin synthesis and terminal erythroid maturation. Mechanistically, 4-HNE-adducted GATA-1 suffers impaired DNA-binding affinity and accelerated proteasomal degradation, thereby crippling erythroid differentiation programs.

### 3.3 Immunomodulatory amplification of erythropoietic suppression

Beyond direct cytotoxicity, Hz indirectly exacerbates anemia by stimulating pro-inflammatory cytokine production (Shio et al., 2010). Notably, natural Hz (containing adsorbed parasite DNA and proteins) usually exhibits stronger immunomodulatory activity than synthetic Hz due to its parasite-derived nucleic acids and protein complexes. Evidence shows that natural Hz can synergistically induces robust secretion of IL-6, TNF-α, and IFN-γ from immune cells (Martiney et al., 2000; Jaramillo et al., 2004; Thawani et al., 2014; Banesh et al., 2022). In contrast, synthetic Hz lacking parasite-derived components exhibits minimal immunogenicity (Thawani et al., 2014). Incidentally, it is suggested that the inflammation status induced by natural Hz is likely to be mediated by activating Toll like receptor 9 (TLR9) (Parroche et al., 2007). Recent studies using human iPSC-derived cerebral malaria models further demonstrate that natural Hz enhances secretion of IFN-γ, IL-1β, IL-8, and IL-16, collectively exacerbating inflammatory anemia (Pranty et al., 2024).

## 4 Direct parasite infection and transcriptional reprogramming

Invasion of bone-marrow erythroid precursors by malaria parasites has emerged as a critical intra-medullary driver of malarial anemia. Using ex-vivo cultures, Tamez and colleagues first showed that the parasite preferentially infects orthochromatic erythroblasts, whereas polychromatic erythroblasts exhibit only sporadic, low-level invasion (Tamez et al., 2009). Microarray profiling revealed that infected orthochromatic cells display altered expression of 609 genes—570 of which are up-regulated—with significant enrichment of NRF2-mediated oxidative-stress pathways and heat-shock proteins (Tamez et al., 2011). Subsequent RNA-seq analyses corroborated the activation of oxidative-stress and mitochondrial-dysfunction pathways, indicating that the parasite re-programmes host metabolism and stress responses to support its own development (Feldman et al., 2023). Co-culture experiments further demonstrated that polychromatic erythroblasts exposed to malaria parasites up-regulate GDF15 (Tamez et al., 2011), a cytokine known to suppress hepcidin and thereby exacerbate ineffective erythropoiesis (Tanno et al., 2007; Guimaraes et al., 2015). Systematic autopsy studies employing dual immunohistochemistry for pLDH and Pfs16 identified the extravascular niche of the bone marrow as the principal site for immature gametocyte sequestration and maturation, with gametocyte fractions reaching 44.9%—far exceeding those in brain (4.8%) or spleen (1.3%) (Joice et al., 2014). Neveu and co-workers (Neveu et al., 2020) subsequently demonstrated that immature gametocytes require approximately ten days to complete stages I–IV within erythroblasts, markedly surpassing the host’s normal enucleation timeline and thus delaying terminal differentiation. Moreover, parasite-infected erythroblasts release extracellular vesicles that elicit oxidative stress and inhibit reticulocyte release (Neveu et al., 2020; Ben Ami Pilo et al., 2022). Collectively, malaria infection establishes a “developmental sanctuary” within the bone marrow by directly infecting terminally committed erythroblasts and deploying paracrine modulators; through transcriptional reprogramming, oxidative-stress signaling is activated and the cell cycle is arrested, thereby disrupting erythropoiesis. Notably, *Plasmodium* infection also profoundly remodels the expression profile of erythroid blood group antigens (Liu and Li, 2024). The EMP3 antigen (Blood Group System 41), whose normal expression is critical for enucleation during terminal erythropoiesis (Thornton et al., 2020), shows aberrant regulation following infection. This dysregulation likely contributes to observed enucleation defects in malaria patients, evidenced by morphological abnormalities in blood smears such as nuclear budding, inter-nuclear bridges, and multinucleated erythroblasts (Brito et al., 2022). This phenomenon may partially explain the impaired reticulocyte production characteristic of malarial anemia.

## 5 Host- and context-dependent heterogeneity in malaria-associated dyserythropoiesis

It should be noted that the mechanisms underlying malaria-induced anemia differ according to host age, nutritional status and clinical contexts, with the relative contribution of each factor mentioned above varying markedly.

In malaria-endemic regions, children under five years of age are likely to experience dyserythropoiesis driven chiefly by inflammatory cytokines such as IL-6, TNF-α and IFN-γ. Their still-maturing immune systems mount vigorous yet poorly regulated pro-inflammatory cascades (Simon et al., 2015) that rapidly suppress erythropoietin synthesis and disrupt the stability of the “master” erythroid transcription factor GATA-1. Moreover, compared with adults, these children possess fewer memory lymphocytes, are more susceptible to infection, and rely more heavily on innate immune responses during the initial phase of infection. A recent study of Malian children corroborates this hierarchy: in 1–5-year-olds, gene expression signatures are dominated by type I interferon, TLR and NLR innate pathways, with minimal T-cell memory signatures, and the proportion of neutrophils rises steeply with parasite density (Tebben et al., 2024). By contrast, older children and adults subjected to repeated low-density parasitemias accumulate hemozoin within splenic and marrow macrophages; persistent oxidative stress then fuels the progressive build-up of lipid-peroxidation products such as 4-HNE, which potently inhibit GATA-1. Consequently, anemia can persist long after peripheral parasitemia has been cleared.

When the host is chronically undernourished, deficiencies in iron, folate or vitamin B12 directly limit hemoglobin synthesis and erythroblast proliferation (Kassebaum et al., 2014). Concurrently, the acute inflammatory response in malaria rapidly elevates hepcidin levels, blocking intestinal iron absorption and macrophage iron recycling (Camaschella, 2019), thereby exacerbating functional iron deficiency and preventing effective erythroid expansion even under EPO stimulation. Moreover, hemoglobinopathies such as sickle cell disease (SCD) or α/β-thalassemia not only alter red-cell susceptibility to *Plasmodium* but also modify the marrow’s “starting burden” and “proliferative ceiling.” (Lelliott et al., 2015) When superimposed on the marrow-suppressive signals of malaria, these conditions can rapidly progress to transfusion-dependent severe anemia (Henrici et al., 2021; Uyoga et al., 2022).

The above factors, such as malnutrition, α/β-thalassemia and SCD, are all distinctly distributed geographically. The Mediterranean-Middle East-South Asia belt is dominated by thalassemia (Tuo et al., 2024), whereas sub-Saharan Africa has the highest prevalence of SCD (Collaborators GBDSCD, 2023). The degree of protection provided by these haemoglobinopathies and their genotypes (homozygous and heterozygous) differs substantially (Taylor et al., 2012). Additionally, the geographic distribution of *plasmodium* species also differs: *Plasmodium falciparum* predominates in sub-Saharan Africa and causes earlier and more severe anemia, while *Plasmodium vivax* is widespread across Asia-Pacific and the Americas and is associated with comparatively milder anemia (Weiss et al., 2025). Therefore, only by integrating the local composite etiologic profile and the prevailing parasite species can precise bone-marrow-targeted interventions be designed.

## 6 Future perspectives

**Figure 1:**
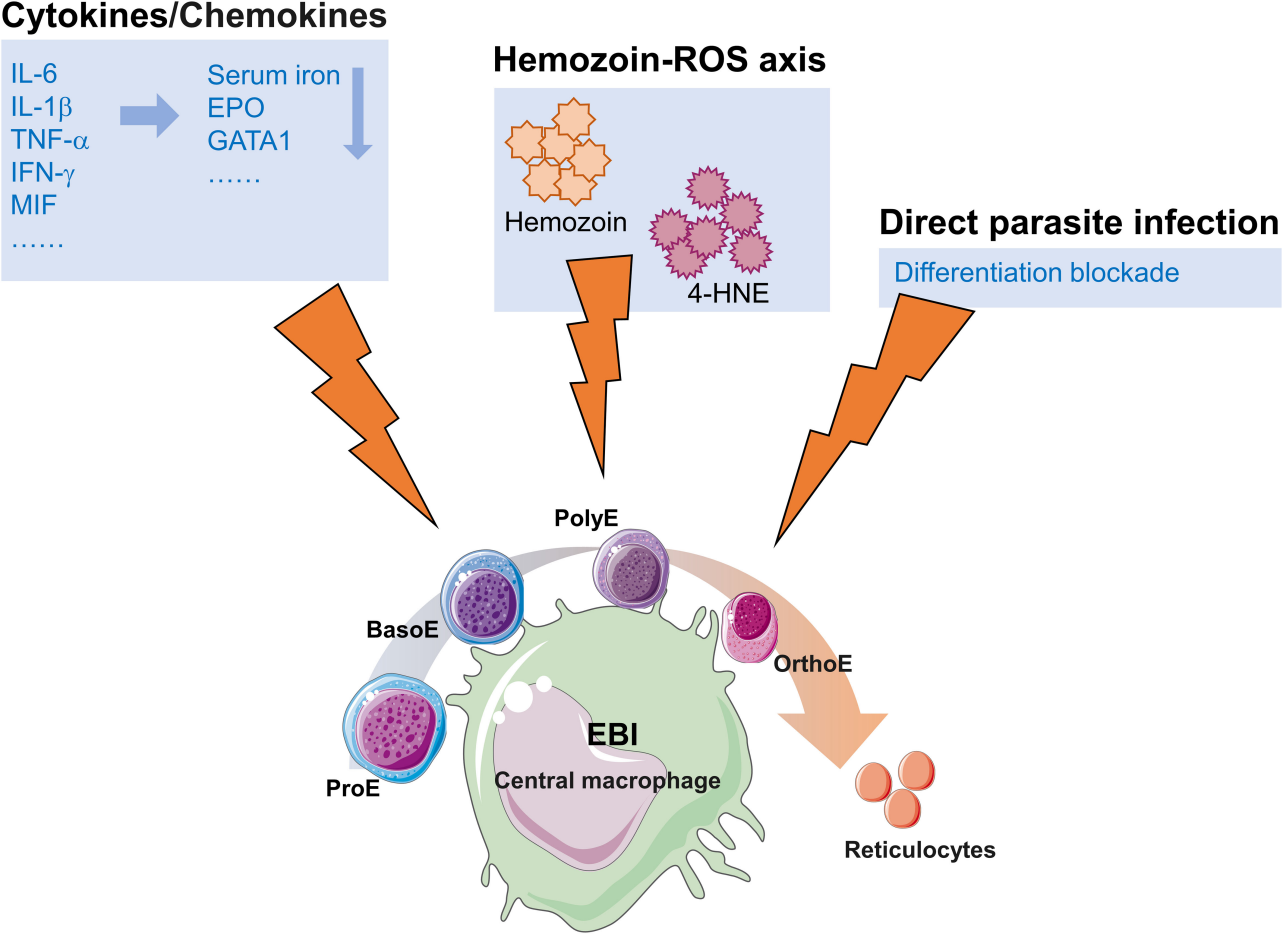
Schematic summary of malaria-induced dyserythropoiesis. EBI, The erythroblastic island; ProE, proerythroblast; BasoE, basophilic erythroblast; PolyE, polychromatophilic erythroblast; OrthoE, orthochromatic erythroblast.

The mechanisms underlying malaria-induced dyserythropoiesis are summarized in **Figure 1**. Despite significant progress in elucidating the pathogenesis of malarial anemia, critical scientific questions remain unresolved. Integration of single-cell technologies with spatial transcriptomics, combined with artificial intelligence-driven multi-omics analysis (e.g., deep learning), holds promise to elucidate the spatiotemporal dynamics of erythropoietic blockade. Spatial transcriptomics preserves tissue spatial conformation, overcoming the technical limitations of single-cell RNA sequencing by enabling single-cell-resolution mapping of parasite-induced transcriptional gradients in the bone marrow microenvironment. Furthermore, the integration of three-dimensional bone marrow organoids with intravital imaging techniques may enable real-time observation of parasite colonization dynamics and host-pathogen interactions within the hematopoietic niche. With advancements in biotechnology, the application of these novel methods and experimental approaches enables further exploration of metabolic dormancy mechanisms in the bone marrow microenvironment and bidirectional signaling between parasites and erythroblasts or other stromal cells. This will deepen our understanding of erythropoietic dysfunction and anemia in malaria. Additionally, ligand-trap agents such as luspatercept (TGF-β/activin inhibition, facilitating late-stage erythroid differentiation) (Martinez et al., 2020; Santini and Consagra, 2025), BRAF inhibitors that treat anemia through activation of MAPK signaling (Wu et al., 2024), and low-cost host-defense peptides with anti-inflammatory properties (e.g., IDR-1018) (Achtman et al., 2012) warrant systematic evaluation as adjunctive therapies for malaria-associated anemia, given their established erythropoietic or anti-inflammatory efficacy in other contexts; such studies may yield unforeseen clinical benefits.
